# Mental Health in the Millennium Development Goals: Authors' Reply

**DOI:** 10.1371/journal.pmed.0040057

**Published:** 2007-01-30

**Authors:** J. Jaime Miranda, Vikram Patel

We appreciate the feedback and responses generated since the publication of our paper of September 13, 2005 [1]. It clearly fulfils our aim of initiating a debate around the core issue expressed in our work—mainly how the targets expressed in the Millennium Development Goals (MDGs) may have the unintended consequence of relegating mental health and noncommunicable diseases in general from the vision of policy makers.

Using evidence on mental health in developing countries, we argued in our paper that addressing mental health problems is an integral part of health system interventions aimed at achieving some of the key MDGs, a view supported by the responses to our paper. However, we should clarify that within the MDGs there is not a “millennium mental health development goal” as suggested by Kasi et al [2].

Sachs and Sachs state that “the reason that the MDGs do not explicitly address noncommunicable diseases such as cardiovascular or psychiatric is because the MDGs focus on the gap in health status of rich and poor countries, a gap mainly accounted for by infectious diseases, malnutrition, and unsafe childbirth. The goals were crafted to address these large gaps rather than to solve all pressing health problems” [3]

We think this assertion deserves their reassessment. It is a common view to assume that the developing world suffers mainly from infectious diseases. In fact, noncommunicable diseases kill people at economically and socially productive ages and kill them mostly in the developing world: 80% of chronic disease deaths occur in low- and middle-income countries [4]. Another misconception is that the epidemic of noncommunicable diseases is still to come. That is no longer true: it is here already [5–7]. We need an informed debate about the health interventions needed to tackle the burden of disease in the developing world, and one that goes beyond the MDGs as they are currently configured.

We are delighted to learn that the Sachs are “exploring ways to integrate mental health care within the health systems,” and agree that development and public health communities—including mental health professionals—need to work together to ensure that the MDGs can be achieved. Explicitly tackling these mental health gaps, in parallel with achieving the existing MDGs, would be a major achievement, resulting in significant improvement in mental health around the world.
